# Civil society participation in the health system: the case of Brazil’s Health Councils

**DOI:** 10.1186/s12992-016-0197-1

**Published:** 2016-10-26

**Authors:** Martha Gabriela Martinez, Jillian Clare Kohler

**Affiliations:** Leslie Dan Faculty of Pharmacy, University of Toronto, 144 College Street, Toronto, ON M4R 1V5 Canada

**Keywords:** Participatory governance, Civil society participation, Civil society inclusion, Good governance, Institutional design, Conselhos de saude, Health Councils

## Abstract

**Background:**

Brazil created Health Councils to bring together civil society groups, heath professionals, and government officials in the discussion of health policies and health system resource allocation. However, several studies have concluded that Health Councils are not very influential on healthcare policy. This study probes this issue further by providing a descriptive account of some of the challenges civil society face within Brazil’s Health Councils.

**Methods:**

Forty semi-structured interviews with Health Council Members at the municipal, state and national levels were conducted in June and July of 2013 and May of 2014. The geographical location of the interviewees covered all five regions of Brazil (North, Northeast, Midwest, Southeast, South) for a total of 5 different municipal Health Councils, 8 different state Health Councils, and the national Health Council in Brasilia. Interview data was analyzed using a thematic approach.

**Results:**

Health Councils are limited by a lack of legal authority, which limits their ability to hold the government accountable for its health service performance, and thus hinders their ability to fulfill their mandate. Equally important, their membership guidelines create a limited level of inclusivity that seems to benefit only well-organized civil society groups. There is a reported lack of support and recognition from the relevant government that negatively affects the degree to which Health Council deliberations are implemented. Other deficiencies include an insufficient amount of resources for Health Council operations, and a lack of training for Health Council members. Lastly, strong individual interests among Health Council members tend to influence how members participate in Health Council discussions.

**Conclusions:**

Brazil’s Health Councils fall short in providing an effective forum through which civil society can actively participate in health policy and resource allocation decision-making processes. Restrictive membership guidelines, a lack of autonomy from the government, vulnerability to government manipulation, a lack of support and recognition from the government and insufficient training and operational budgets have made Health Council largely a forum for consultation. Our conclusions highlight, that among other issues, Health Councils need to have the legal authority to act independently to promote government accountability, membership guidelines need to be revised in order include members of marginalized groups, and better training of civil society representatives is required to help them make more informed decisions.

## Background

Institutional reforms within the international development field have increasingly focused on establishing *civil society participation* in decision-making and policy formulation to promote social justice and increase good governance in the public sector [1]. In our paper, we use the term “civil society” to refer to “ordinary citizens,” who are independent of the government. We also use the term “participation” to describe a process by which civil society and government come together to engage in active dialogue to take a decision in a collaborative manner [2, 3].

The recent Sustainable Development Goals (SDGs) recognize that, “responsive, inclusive, participatory and representative decision-making at all levels” is a crucial component for development [4]. Governments commonly create a forum or space in policy making, through which civil society can voice demands and concerns. Ideally, the inclusion of civil society raises the potential for decision-making to be well informed and reflective of societal needs [5, 6]. This, in turn, can make civil society more supportive of government decisions and increases (either real or perceived) government support and legitimacy [5, 6]. The inclusion of civil society in the decision-making process can also increase transparency by helping inform citizens about how decisions that affect their everyday lives are made [5]. Transparency is linked to accountability in that government transparency requires that citizens be fully informed about how and why decisions are made, including the decision-making procedures followed, criteria applied by policy-makers, and the information or evidence drawn upon to reach decisions [7]. This information can then be used to hold relevant government officials accountable to their actions to ensure satisfactory public sector services [8, 9]. This can also help monitor the performance of the health system and resource allocation in the public sector [10–12].

Globally, there are many examples of forums for civil society participation in the health sector; many of these have been established in the past two decades. For example, the Province of Quebec’s Hospital Boards, that include civil society in the decision making process in Quebec’s hospitals, the United Kingdom’s Public Participation Forums that include civil society in the allocation of resources of the United Kingdom’s National Health Service, and Brazil’s Health Councils, the latter is the focus of this paper.

Civil society participation in the health sector assumed a central role in the restructuring of Brazil’s unified health system, the *Sistema Único de Saúde* (SUS), which took place during the country’s period of transition out a military regime during the 1980s. This focus on civil society participation grew out of the *Sanitarista* movement, which advanced the decentralization of Brazil’s health system and universal health coverage for the population. This same social movement promoted the establishment of Health Councils in 1990 to provide a forum for civil society participation in the implementation and monitoring of health policies for social accountability (*control social* in Portuguese) at the municipal, state and federal levels [13–15]. Today, these councils serve as advisory bodies with an ambitious mandate that includes monitoring the health system and the allocation of resources, bringing together civil society groups, heath professionals, and government officials.

The 48 members of any given Health Council are represented as follows: 50 % are ‘users’ of the healthcare system (civil society groups), 25 % are healthcare representatives and 25 % are government representatives. Government representatives may be appointed by the following: the National Council of Secretaries of Health (CONASS), the Federal Government, the National Council of Municipal Health Secretaries (CONASEMS), healthcare service providers, and lastly, private sector representatives. Civil society and healthcare representatives, on the other hand, are appointed to Health Councils by their respective organizations/institution. The creation of the Health Councils is considered to be a milestone in the history of health policies in Brazil; Health Councils have also received international praise for their capacity to advance participatory democracy [16].

Still, there is not always clear evidence of what impact civil society has had on policy within forums like the Health Councils. Indeed, in reference to efforts by its own member states, the Organization for Economic Co-operation and Development (OECD), has noted that, “… *there is a striking imbalance between the amount of time, money and energy that governments in OECD countries invest in engaging citizens and civil society in public decision-making and the amount of attention they pay to evaluating the effectiveness and impact of such efforts*” [17]. Studies of different models for civil society participation in the health policy process have illuminated some of their limitations. For example, a study conducted on British Columbia’s Regional Health Boards found widespread feelings of discontent among civil society members about their role within the Boards, as the government failed to clearly articulate during their formalization why these Boards were being created or how civil society was to be included in decision-making [18].

In the case of Brazil, much of the existing literature on Health Councils focuses on their prevalence throughout the country, as well as the status that they hold, which gives them the* potential *to serve as a deterrence mechanism for corruption and also as a way to increase social accountability by the government in the provision of health services [19]. Yet, there is evidence from some studies that that certain aspects of the Health Councils, such as meeting guidelines and council membership procedures, have unintended created barriers for civil society engagement in health policy formulation [13, 20–26].

To be sure, a challenge in any effort to include civil society in the policy process is first to determine who should represent civil society. Often terms such as ‘citizens’, ‘users’, ‘civil society’, and ‘the public’ are used interchangeably across different models without a clear definition of who exactly they are referring to and thus whom these forums aim to include [27]. This is particularly important for the health sector given the mix of technical and non-technical knowledge necessary for making decisions for health policy formulation [9]. On a similar note, terms like ‘engagement’, ‘participation’ and ‘involvement’ are used often without an explicit explanation of the degree to which civil society will be engaged in decision-making or the roles and expectations of civil society participants [6, 27, 28]. Governments rarely specify how and when civil society’s views and demands will be included in the policy and decision-making process [29]. This lack of clarity suggests the need to understand better the strengths and weaknesses of how current participatory policy models are designed so that future efforts incorporate lessons [9, 30]. To this end, we have explored one of these models – Brazil’s Health Councils.

## Methods

### Semi-structured interviews

#### Data collection

A total of 40 semi-structured interviews were conducted with Health Council Members at the municipal, state and national levels by the first author and a Research Assistant. In June and July of 2013, the first author and a Research Assistant interviewed eight municipal, seven state, and six national Health Council members. In May of 2014 a Research Assistant interviewed an additional nine municipal, nine state and one national Health Council members. The geographical location of the interviewees covered all five regions of Brazil (North, Northeast, Midwest, Southeast, South) for a total of 5 different municipal Health Councils, 8 different state Health Councils, and the national Health Council in Brasilia. Due to confidentiality concerns, the location of study participants cannot be disclosed. Table 1 provides a breakdown of the study sample.

**Table 1:** Breakdown of study sample

	Municipal Level	State Level	National Level	Total
Civil Society Representatives	8	9	4	21 Civil Society Representatives
Health Sector Representatives	7	4	1	12 Health Sector Representatives
Government Representatives	2	3	2	7 Government Representatives
Total	17 Municipal Health Council Members	16 State Heath Council Members	7 National Health Council Members	40 Health Council Members

Study participants were identified by accessing membership lists of Health Council at the national, state and municipal levels which were available online. We began with the membership list of Brazil’s national Health Council, followed by searching for state Health Councils that had a membership list available online. We then identified the municipal Health Councils by using state Health Councils’ websites that had links to municipal health councils’ websites in them. We invited all Health Council members on the available lists through an email invitation, for a total of about 1,400 invitations. When a phone number was available, non-respondents were contacted a second time via telephone to solicit a response. Due to the low number of respondents reached in this manner, we also used snowball sampling. Written consent was received from each interviewee prior to the interview. Interview questions (Appendix 1) were formulated based on issues highlighted in existing literature on civil society participation in Brazil and focused on their views and perceptions of interviewees’ respective Health Councils. The interviews were conducted in Portuguese over the phone and Skype. The 2013 interviews were not tape-recorded but two note takers captured the content of the interviews. The 2014 interviews were tape recorded, transcribed and translated from Portuguese to English with the support of a Brazilian researcher. The first set of interviews was not tape-recorded because the study participants did not consent to being tape-recorded. In order to address any loss in meaning due to translation, all of the interviewee quotes in this paper are provided Portuguese in Appendix 2.

#### Data management and analysis

Data was coded using an inductive approach and themes were generated using a thematic approach. We first read through the interview content to generate a preliminary list of codes using inductive analysis. Then, we coded the interview data using inductive analysis with the software HyperResearch. We created a final list of codes by selecting the codes that were most relevant to the research question. These codes were then grouped together based on their relevance to one another to form themes. The data extracts for each theme were reviewed to create a detailed summary (with quotes) that captured the main messages from each set of extracts. At this stage differences between civil society representatives, healthcare representatives and government representatives within each theme were identified by the researcher and noted in the theme summaries. These summaries were then used to help present the themes in the results section. The analysis conducted did not focus on identifying underlying assumptions and ideas that gave form to interviewees’ views and perceptions in the data collected.

### Legislative review

Once interviews were analyzed and themes were identified, a scan of the decrees, resolutions and amendments that are relevant for Health Councils was conducted, using the National Health Council as the reference point for Health Councils at the state and municipal levels. This scan only focused on documents that related to the issues identified through the interviews. For example, if one of the identified themes was ‘issues with membership guidelines’, only legal documents pertaining to Health Councils’ membership rules were considered. This was done in order to corroborate the findings from the interviews conducted. Table 2 outlines the documents reviewed.

**Table 2:** List of documents reviewed

Document	Description
Resolution No. 291, 6 May 1999	Outlines what each of the three types of Health Council decisions are (motions, recommendations, resolutions)
Resolution No. 407, 12 September 2008	Outlines the overall design of Health Councils
Decree No. 4839, 11 July 2006	Provides greater detail on membership guidelines
Resolution No. 453, 10 May 2012	Provides greater detail on the Health Council budget

### Ethical considerations

This study was reviewed and approved by the University of Toronto’s Research Ethics Board (Protocol Reference # 28320) as well as Brazil’s National Commission of Ethical Research (*Comissão Nacional de Ética em Pesquisa – CONEP*) (Approval # 686.734).

## Results

The study findings are organized around the following themes: 1) Lack of autonomous authority; 2) Membership guidelines that limit inclusion; 3) Government pressure on Health Council members; 4) Lack of support and recognition from government; 5) Insufficient resources for Health Councils; and, 6) Health Council members’ strong individual interests.

### Lack of autonomous authority

Brazil’s Health Councils were established to monitor both the public and private health sectors, to monitor and approve the country’s health budget and its allocations, and to assist in the implementation of the national health policy. Decisions taken in Health Council meetings are divided into motions, recommendations, and resolutions. Motions are used to express recognition, support, and criticism of a specific subject, and they are non-binding. Recommendations are, on the other hand, suggestions, warnings, or notices about specific issues but they are also non-binding. Health Councils also can put forward resolutions that can be implemented into law, if and only if, the Ministry of Health approves them. Therefore, Health Councils do not stand as autonomous bodies independent of the government and have no legal authority through which they can ensure that their resolutions are implemented.

Health Council members at the national, state and municipal levels reported that the decisions taken during meetings are not followed up well. A Health Council member at the state level reported,

“A resolution that had been passed in the Council about abolishing the privatization of the health system was shut down by the last two Health Secretariat of my state. One of them said he wasn’t going to ‘shoot himself in the foot’ by implementing it.”^1^ (Civil Society Representative, State Health Council Member #2)

It seems that civil society’s participation in Health Councils is more consultative in nature, as the government holds the ultimate decision-making power because it has the legal authority to implement decisions. This dependency on the government to implement Health Council decisions also means that there is no guarantee that civil society’s input will be part of the final decision-making process, as this is solely dependent on the given government’s willingness to take their input into account. This has not only created a sense of frustration among Health Council members, but it also puts into question how effectively Health Councils’ can fulfill their mandate, given that they cannot take action to hold governments accountable without, somewhat paradoxically, having that same support from the government.

### Membership guidelines limit inclusion

Health Councils’ membership guidelines were designed to include government officials, civil society representatives (referred to in Portuguese as *usuarios*, or users of the healthcare system), and healthcare representatives. Fifty percent of any Health Council must be composed of civil society representatives, which are defined as “representatives of organizations and social movements that have expertise and representation in at least one third of the units of the Federation and three geographical regions of the country for at least two years” [31]. Twenty-five percent of membership seats must be filled by healthcare representatives, which are defined as “representatives of health professional organizations, including the scientific community” [31]. The other 25 % is to be comprised of government representatives from the National Council of Secretaries of Health (CONASS), the federal government, the National Council of Municipal Health Secretaries (CONASEMS), healthcare service providers, and business entities that conduct business within in the health system [31]. Civil society representatives are appointed by their respective organization and an independent committee must elect each organization every three years. No minimum technical qualification is required from civil society representatives to participate. However, membership guidelines are limiting in scope, as only organized groups that have a strong presence throughout Brazil and are formally recognized by the government can qualify to participate.

A reported lack of interest from civil society in participating in Health Councils has resulted in limits to the breadth of the membership. Interviewees at all three levels of government, underscored that the public is often unaware of the existence of Health Councils, and those who do have some knowledge about Health Councils, are often not interested in learning more about what they do or how to participate within them. A Health Council member representing civil society at the municipal level stated,

“Civil society participation is very fragile. Our society doesn’t have a culture of participation in the creation of public policies, and in the 21^st^ Century they don’t realize the power that it has by being a taxpayer, a citizen with rights. Society in general is unaware of that the State exists to give back the high taxes that are levied and collected in the form of services like education and health. Brazilian society is taking too long to wake up.”^2^ (Civil Society Representative, Municipal Health Council Member #8)

This suggests that civil society needs to have a better understanding about Health Councils and how they can effectively participate in them. Coupled with restrictive membership guidelines, Health Councils do not necessarily represent the ‘users’ of the healthcare system well. Rather, membership tends to be limited to well-resourced and organized members of Brazil’s population. This leaves behind poor-resourced populations that use SUS’s services but lack the necessary time and money to be organized and qualify to participate. Health Councils’ breadth of population inclusion is therefore narrow in scope.

### Government pressure on health council members

Health Councils members at all three levels reported feeling “pressured” by their respective government representatives; thus affecting their ability to work impartially and openly. Health Council members stated that government representatives often used bribery and intimidation tactics to sway member decisions and how they voted. At the national level, a Health Council member expressed that, “…in order to get things done you need to be good friends with the government.” (Government Representative, National Health Council Member #1) Another Health Council member at the state level reported that members are “constantly being manipulated by government representatives” (Healthcare Representative, State Health Council Member #1) through favor exchanges such as job offers or punishments through lay-offs and threats. Members are often threatened or pressured to take decisions that will not create a negative image for a given government. Not surprisingly, this has reportedly created a hostile environment during Health Council meetings.

A Health Council member representing civil society at the state level reported,

“There is a legal framework. We try to work impartially, defending the interests of the public [pause] but the government pressures us, especially when it comes to decisions that have to do to with the health budget. Health workers are the most vulnerable ones.”^3^ (Civil Society Representative, State Health Council Member #11)

Another Health Council member representing the health sector at the state level stated,

“Civil society representatives are vulnerable to the government’s interests. Government representatives, as one might expect, support almost blindly the interests of the government. These interests are often contradictory of those of civil society. They will do anything under their power to avoid embarrassing situations for the government. Health representatives act pretty much the same way… they show a real disconnect from the real needs of society. It seems that there is no real commitment to meet societal needs.”^4^ (Healthcare Representative, State Health Council Member #8)

Health Council members are thus often reluctant to engage in open discussion due to possible retaliation by the government. A Health Council member representing civil society at the municipal level reported,

“We do what we can. Sometimes, we personally go and talk to patients, doctors. Other times, we write to them to reach out. Laws are there to be followed but sometimes we do not report issues because of fear. Council members work for or may have relatives who work for the government and we are afraid to lose our jobs or our position in the council. It’s really a shame. If there were equal rights and laws were enforced, our Brazil would be a model to follow.”^5^ (Civil Society Representative, Municipal Health Council Member #12)

Another Health Council member representing the healthcare sector at the municipal level reported that,

“The Health Council that I belonged to always works with the information available and decisions are taken according to the law. Sometimes, the government is uncomfortable with that, mainly when it comes to embezzlements. The government threatens some Health Council members, especially those working for the government. If a Health Council member works for a private company and goes against the government during Health Council meetings, the government will intimate them by asking for an inspection of their company. Health Council members feel threatened because according to the law, the Health Council will be punished if it approves any process in which resources are suspected to be stolen or misuse. That is why some members prefer to leave the Health Council. Their participation is voluntary, they are not paid for that, and it takes time to participate, oversee how the budget has been used and investigate civil society’s accusations of irregularities in the health care system. I don’t blame them.”^6^ (Healthcare Sector Representative, Municipal Health Council Member #15)

These accounts suggest power imbalances among council members; it seems that government representatives have the greatest power over civil society and healthcare representatives, putting the latter groups in a vulnerable position. This can clearly have an influence on what is discussed during Health Council meetings and the type of decisions that are taken as a result of the discussions. Ultimately, power imbalances limit the Health Council’s ability to effectively monitor the healthcare sector, given that they have no authority over the same government they aim to monitor and hold accountable to the public.

### Lack of support and respect from the government

Healthcare and civil society representatives at all three levels of government also stated that there is a lack of support from their respective governments (e.g. national, state or municipal). Not surprisingly, governments will support health councils and implement their decision only if they are aligned with their own agenda. A Health Council member representing civil society at the municipal level stated,

“The government decides when to interfere with partisan politics. Sometimes they do more than they need to, but most of the time they just do what is required of them.”^7^ (Civil Society Representative, Municipal Health Council Member #12)

A Health Council member representing civil society at the national level stated,

“The government does not really support the councils, it tolerates them. There is tension between the two because of the differences in interests. It is more of an instrumental relationship. They don’t realize the power that Health Councils have. The government is more concerned with the opinion of government representatives than those of civil society or healthcare representatives, maybe because other interests are involved.”^8^ (Civil Society Representative, National Health Council Member #6)

Health Council members expressed that their respective government is generally not interested in having Health Councils work effectively in case they create a threat to their power. A Health Council member representing civil society at the municipal level stated,

“The government does not recognize the Council as an autonomous body and has no real interest in making sure it’s working properly. The State Health Council conducts trainings, meetings, but the most important thing is encouraging civil society to participate, but the government is afraid of making this happen because they don't want to lose the power they believe is theirs and not of the people who elected them.”^9^ (Civil Society Representative, Municipal Health Council Member #8)

At the state and municipal levels, this lack of recognition and support from their government sometimes results in a government failing to address issues that are critical, within the jurisdiction of Health Councils, such as the approval of health budgets. A municipal Health Council member representing civil society stated,

“For the last 4 months, the government has failed to provide us with the budget reports that need to be approved by the Health Council. We are often not consulted on issues that correspond to us and things get approved without our consent even though it is needed under the law.”^10^ (Civil Society Representative, Municipal Health Council Member #12)

As noted earlier, Health Councils will be effective only if their respective government decides to do so. The lack of power or legal authority Health Councils holds has meant that they have largely not been able to have a real impact on the policy process.

### Insufficient resources for health councils

#### Budget

Pursuant to Resolution 453/2012, taken by the National Health Council, the three levels of government must provide the necessary financial and administrative resources for their respective Health Councils to have administrative autonomy to function effectively. For example, the respective government of any given Health Council is required to provide a physical space so that Health Councils are able to hold meetings and financially support administrative staff and promotional materials. The Ministry of Health, in conjunction with the Federal government, is charged with working with the state and municipal governments to ensure the funds are transferred and provided to the Health Councils. However, in practice, at the state and municipal levels, there are budget constraints have limited Health Council meetings and their ability to disseminate information relevant information to the public. A Health Council member representing civil society at the state level shared that,

“The government doesn’t support us a lot but our existence is guaranteed by the law. Once in a while the Internet is suspended because the government doesn’t pay it on time. When this happens we use our cellphones and make things work, even with these challenges.”^11^ (Civil Society Representative, State Health Council Member #2)

Another Health Council member representing civil society at the state level reported that,

“The government says they support the councils but it’s far from reality. We don’t have our own website to disseminate information to the public. The website we have we paid for out of our own pockets and it doesn’t support a lot of data..”^12^ (Civil Society Representative, State Health Council Member #5

In addition, Health Council members also reported that the management of their budgets lack transparency and noted that there examples of budget mismanagement by government officials who are in charge of it. At the state and municipal levels, Health Council members reported that their budget allocation is rarely discussed during meetings. Also, at the municipal level, Health Council members reported that there is no planning process regarding how the budget is to be spent during the year, which results in the budget not being used or mismanaged.

#### Training

Under the *National Policy of Permanent Education for Social Accountability in the Health System *(*Política Nacional de Educação Permanente para o Controle Social no Sistema Único de Saúde*), training for Health Council members is a mandated requirement. This policy expresses further that municipal, state and federal Secretaries of Health must provide funding to train Health Council members on the structure of the health sector, their mandate and any relevant laws and policies. However, it does not explicitly outline how training is to be provided, how long it should take place, nor does it provide a curriculum that explicitly states what needs to be taught to Health Council members. This ambiguity may explain why many Health Council members reported feeling unprepared and unable to engage in discussions during meetings. This lack of training was further supported by healthcare representatives at all three levels of government who stated that civil society representatives are unable to engage in active discussions on the health sector due to their limited knowledge. For example, a healthcare representative at the state level reported,

“Some Health Council members are not prepared to engage in discussion. The training provided is definitely not enough for them.”^13^ (Healthcare Sector Representative, State Health Council Member #7)

The inadequate allocation and management of the operational budget of Health Councils, as well as poorly trained members, have contributed to weakness of Health Councils. Health Council members need to have a solid foundation of knowledge about how the health system and Brazil’s legal system works, as well as their rights as Health Council members and the government’s obligations to them.

### Health council members’ strong individual interests

Evidently, there are Health Council members who are pursuing their own interests over and above those of the institution they represent. There was indeed wide agreement among interviewees that Health Council members often advance their own private interests. A Health Council member representing the government at the municipal level stated,

“Does the council work in an impartial way? For me, that is the main problem of Health Councils. Unfortunately, they were created to mobilize civil society into SUS, but it did not work. People are there to defend their own interests or the interests of the institutions they represent, with some exception. For example, I have been participating in different management positions of this Municipal Health Council over the years, and depending on who is in the management position, the agenda of the Secretariat changes … There was a management period that was focused on mental health. My Goodness, we had to be careful to present anything related to mental health … In another time, the management was focused on dental health. Currently, this management focus is on workers’ health.”^14^ (Government Representative, Municipal Health Council Member #9)

There is also a reported apathy among some members. A Health Council member representing civil society at the state level reported that,

“Some civil society groups have ties to the Health Councils, but they don’t care about [Health Councils]. They send people to represent them who do not know why they are even there.”^15^ (Civil Society Representative, State Health Council Member #5)

Another Health Council member representing civil society at the municipal level stated that,

“A big issue that I see is that most of the council members don’t like to read, work all day and sometimes this limits their ability to act. There is a lot of information on laws, resolutions, and regulations.”^16^ (Civil Society Representative, Municipal Health Council Member #8)

While some of members interviewed reported having a genuine interest in improving the overall state of the health system, they also reported that many of their peers on the Council did not share this goal.

## Discussion

The findings of this study illuminate some of the challenges present in Health Councils that limit their effective functioning and the capacity of civil society to have a meaningful input into the health policy process. One clear limitation is the absence of a legal framework that provides Health Councils with the authority to ensure accountability of their respective government and to enforce the rule of law. Government representatives, it seems, have greater incentives to dominate and even manipulate discussions and decisions in Health Councils, since their livelihood and power is directly tied to their outcomes [27]. Meanwhile, civil society’s participation seems to be largely limited to well-resourced groups so that the most marginalized populations are, in fact, not represented in the Health Councils. We found that in many Health Councils, government representatives often use manipulative tactics. When they are unable to “buy” support from other members, they have allegedly resorted to threats, particularly towards healthcare representatives. This patently fosters a climate, whereby many Health Council members will be reluctant to voice their real demands and concerns. Indeed a study conducted by Cornwall (2008), highlighted that given the hierarchical nature of the healthcare system and their reliance on the government for work contracts, healthcare representatives feared being fired for voicing their concerns and opinions [23]. As a result, government representatives have seem to share the greatest influence Health Council meetings, which puts into question how well participatory democracy works within them.

The apparent power asymmetry between civil society and government representatives present in Health Councils is further magnified by the reported lack of training that is provided to Health Council members. Due to the complexity of the healthcare sector and the policymaking process, civil society representatives need to possess a sufficient level of technical knowledge for informed discussion about health sector policy issues. We found training is insufficient, which has had a reported negative effect on civil society representatives’ ability to engage in active and meaningful discussions and make informed and evidence-based decisions. Within Health Councils, civil society is generally less equipped to participate in discussions, compared with healthcare and government representatives, who have greater technical knowledge [22].

Additionally, the membership guidelines of Health Councils are not conducive to broad membership of the population, as they are best suited for well-organized and active civil society groups. Marginalized groups of Brazilian society need to be represented as they are most likely to depend the most on the healthcare services provided by SUS due to their financial inability to turn to the private sector [32]. Socioeconomic factors such as income and educational levels contribute to which members of the population represent civil society within Health Councils [6, 22]. The membership guidelines effectively create a restrictive level of civil society inclusion, whereby only those who are willing to participate and have the means to form an organized civil society group can qualify for membership. A prior study on the State Health Council of São Paulo also concluded that membership guidelines create “a pre-existing network of relationships among representatives of government and social movements” and exclude those that lack the means to form such ties [13].

The low level of participatory culture that can be found in Brazil has had a reported effect on civil society’s level of participation and interest in Health Councils. And even when civil society participates, representatives may only use Health Councils to advance their own interests. Many members use their membership as a stepping-stone for a government career or as a mean to gain prestige among peers [23].

Other studies conducted on Health Councils in Brazil have also highlighted their limitations. For example, one study reported that Health Council members felt that they have failed to have a meaningful effect on health policies [22]. Another study concluded that while multiple issues in the healthcare system are raised within Health Council discussions, they have failed to influence any decisions taken by the government [13]. And lastly, even though Health Councils permit the inclusion of new actors in health policy discussions, they have not had a significant effect on the restructuring of the SUS [26].

If Brazil’s Health Councils are to become a meaningful forum for inclusive health policy discussion and outcomes, a number of changes are likely needed. First, Health Councils need greater authority and autonomy from the government. Second, membership guidelines need to incentivize participation amongst all members of the population and specifically make greater efforts to have provisions that will include marginalized groups. This will ideally yield greater levels of civil society participation and help ensure that marginalized group issues and concerns are factored into the decision-making process. Third, the Federal government must clearly outline how civil society’s input will be incorporated in the decision-making process in order to ensure policy that is reflective of societal needs, healthcare representatives’ expertise, and the governments’ knowledge of policy making. This will help promote more transparency in the decision-making process and manage expectations among Health Council members better. Lastly, clear training guidelines and sufficient curricula, along with the requisite resources, are needed to ensure sufficient training is provided to Health Council members, particularly those from civil society. Training will give civil society representatives the requisite knowledge to actively engage in discussions and health sector policy processes.

## Limitations

 The authors analyzed the interview data with the assumption that participants’ accounts are purely objective. This therefore led to the generation and analysis of data that does not take into account participants’ beliefs and values and how those shaped the study’s reported findings. Therefore, future studies can address this limitation by using different ontological assumptions to help understand how these conditions and context may have had an effect on this study’s findings.

In addition, our decision to conduct interviews with Health Council members was based the study’s objective of creating a descriptive account of issues present in this specific forum for civil society participation in decision-making. However, despite the researchers’ best efforts, the response rate from government representatives was low, which created an overrepresentation of healthcare professionals and civil society representatives in our sample. This may explain the predominance of issues surrounding government support in our results. Future work on Health Councils could explore government representatives’ views and perceptions in more detail to have a more balanced recount of some of the issues present in Health Councils from the perceptions of all the parties involved. In addition, while our findings may be similar to studies conducted on other models of participatory governance, they are not representative of all forms of participatory governance models. Therefore, transferability of our research findings must be mindful of the context under which this research was conducted as well as the researchers’ assumptions that guided the data analysis process. Lastly, despite our best efforts, the authors acknowledge that the analysis of the interview data may have been affected during the translation process in a way that certain words or phrases may have been “lost in translation”. This was mitigated as much as possible by actively consulting with a Brazilian researcher fluent in both English and Portuguese on the appropriateness of word selection and meaning.

## Conclusions

This study highlighted how Brazil’s Health Councils have not necessarily led to meaningful participation by civil society in the health policy process. They certainly provide a forum for stakeholders to come together to discuss issues in the health sector and they have the potential to make members of civil society feel empowered and better informed about the healthcare sector. However, we found that they do not necessarily provide a sufficient forum through which civil society can actively and meaningfully participate in the decision-making process. This puts into question how well participatory democracy is served by the Health Councils.

We found that civil society is limited in Health Councils throughout Brazil by restrictive membership guidelines, a lack of autonomy from the government, vulnerability to government manipulation, a lack of support and recognition from the government, and a lack of necessary training and budget [6, 29]. As a result of these issues, Health Councils may not be an effective forum through which civil society can engage in discussion to promote policy that is reflective of societal needs. There is certainly no one size fits all model to achieve this. However, this study has shed light on the need for Health Councils to have more defined terms and goals and to have its mandate backed up by a strong and independent legislative framework to achieve it. This would help guide policy makers’ decisions on who should be included, how their views will be included in decision-making, and when civil society should be included. Health Councils would also benefit from having the legal authority to act independently in order to minimize vulnerabilities to government manipulation. Lastly, membership guidelines ideally should be revised to ensure greater inclusion that does not rely on the organizational capabilities of civil society groups and better training for civil society representatives to take more evidence-based decisions.

## Appendix 1

### Interview guide

Please introduce yourself and state which group you present on your Health Council?

How long have you been involved in the council? Why did you join?

Do all council members actively engage in discussions? Why or why not?

How would you describe the relationships between council members?

How does your Health Council communicate with the public? Is it effective? Why? Why not?

How do citizens participate with the Health Councils?

Is the budget used effectively? Who is in charge of it?

Is the way in which meetings are run effective? Why or why not?

What type of training council do members have? Is it enough?

Are Health Council decisions implemented by the government? Give examples.

Do Health Councils have any power to enforce any of their decisions?

Does the government support Health Councils? How? Or if no, why not?

What is the biggest strength/weakness of the councils?

What improvements would you make?

## Appendix 2

### Quotes from interviews in Portuguese

^1^“Uma resolução que foi aprovada no Conselho contra a privatização do sistema de saúde não foi assinada pelos dois Secretários de Saúde anteriores do meu estado. Um deles disse que não ‘ia dar um tiro no próprio pé’.”

^2^“A participação da sociedade civil e muito frágil. Nossa sociedade não tem a cultura de participação na construção das políticas públicas e, ainda, em pleno século XXI não se deu conta do poder que possui por ser contribuinte, cidadão de direitos. A sociedade em geral desconhece a finalidade da existência do Estado em devolver-lhe sob a forma de serviços como educação e saúde, os altos impostos que são cobrados e recolhidos. A sociedade brasileira está demorando muito a despertar.”

^3^“Existem leis. A gente tenta trabalhar com imparcialidade, defendendo os interesses do público [pausa], mas há pressões do governo, especialmente quando se trata de decisões sobre o orçamento da saúde. Os trabalhadores de saúde são definitivamente os mais vulneráveis.”

^4^“Os usuários são vulneráveis aos interesses do governo. Os gestores, como esperado, apoiam o governo com cegos. Esses interesses não são idênticos aos da sociedade. Eles vão fazer qualquer coisa para evitar escândalos. Os trabalhadores de saúde atuar da mesma forma … suas ações não refletem as necessidades da sociedade. Parece que não há nenhum compromisso real para atender as necessidades da nossa sociedade.”

^5^“A gente fazem o que podem. Às vezes, falamos com pacientes, médicos. Outras vezes, escrever-lhes. Leis existem para ser seguidas, mas às vezes temos medo de reportar problemas. Os membros do Conselho trabalham para ou pode ter parentes que trabalham para o governo e nós estamos com medo de perder nossos empregos ou nossa posição no conselho. É realmente uma vergonha. Se houvesse direitos e leis foram aplicadas, nosso Brasil seria um modelo a seguir.”

^6^“O conselho de saúde que presidia sempre trabalha com as informações e depois de analisadas e que poderá afetar um lado ou outro, mas afirmo que as decisões serão tomadas conforme a legalidade e isso aplicará certo desconforto ao gestor, principalmente nos desvio de recurso que a tomada de decisão reprovada pelo conselho, faz com que este procedimento gera perseguição a conselheiro que é funcionário público e também da iniciativa privada que tem empresas e é perseguida por mandato de fiscalização para intimidar as ações aplicadas que a contrarie a sua gestão. Os conselheiros se sentem ameaçados, por que, a legislação joga a responsabilidade no conselho, o mesmo responde criminalmente aprovar qualquer procedimento que envolva recurso que haja suspeita de fraude ou desvio. A onde em que cada membro prefere sair do conselho por que é uma participação voluntária e não ganha nada com isso e toma tempo para dedicar às atividades das comissões nas fiscalizações dos recursos, ações e denuncias dos usuários do sistema de saúde. Não os culpo.”

^7^“Os gestores decidem quando fazer interferencia da política partidaria. Muitas vezes podem fazer mais, outros fazem o que são mandado. ”

^8^“Na realidade, o governo não apoia os conselhos, eles os toleraram. Há uma tensão entre os dois devido às diferenças de interesses. É mais uma relação instrumental. Eles não percebem o poder que os conselhos de saúde têm. O governo se preocupa mais com a opinião de seus representantes do que os usuários ou profissionais de saúde, talvez porque outros interesses estão envolvidos.”

^9^“O governo municipal não reconhece o Conselho como órgão autônomo, deliberativo, não tem interesse real em qualificá-lo. O Conselho Estadual realiza capacitações, reuniões, orienta, mas o cerne da questão seria incentivar a participação social e, isso, os administradores públicos não fazem com medo de perder o poder que acreditam ser deles e não do povo que pediu para representá-lo.”

^10^“Por os últimos 4 meses, o governo não nos deu os relatórios orçamentais que devem ser aprovados pelo Conselho. Muitas vezes não nos dizem coisas que devemos aprovar e as coisas são aprovadas sem nosso consentimento, mesmo que é exigido antes de a lei.”

^11^“O governo não nos apoia muito, mas nossa existência é garantida por lei. No entanto, de vez em quando a internet fica suspensa por falta de pagamento do governo estadual aos fornecedores. Mas nós usamos nossos celulares e fazemos acontecer mesmo com dificuldades.”

^12^“O governo diz que apoia os conselhos, mas está longe de ser realidade. Eles não têm o seu próprio website para divulgação de informações. O site que existe a assessoria de comunicação paga com dinheiro do próprio bolso. Ele não suporta muitos dados. Há falta de apoio do governo.”

^13^“Alguns conselheiros não estão preparados para se envolverem em discussões. A formação ministrada definitivamente não é o suficiente para eles.”

^14^“Funciona de forma imparcial? Para mim esse é o principal problema do CMS, infelizmente foi uma forma inventada para o SUS de mobilização da sociedade que não deu certo. As pessoas estão lá para defender interesses próprios, salvo exceção, interesses das instituições que representam. Exemplo disso, já passei por algumas gestões do CMS e dependendo de quem está lá o foco de cobrança para a Secretaria muda… Houve uma gestão que foi saúde mental, minha nossa, tínhamos o maior cuidado para apresentar qualquer coisa de saúde mental.. outra era Saúde Bucal. Nessa gestão o foco é saúde do trabalhador….”

^15^“Algumas entidades têm cadeiras no conselho, mas não se importam. Mandam pessoas que não sabem o que estão fazendo lá.”

^16^“Um grande problema que vejo é que a maioria dos conselheiros não gosta de ler, trabalha o dia inteiro e, às vezes, sofre restrições para poder atuar. Mas há muita informação nas leis, nas resoluções e portarias.”

## Abbreviations

CONASEMS: Conselho Nacional de Secretários Municipais de Saúde, Brazil’s National Council of Municipal Health Secretaries
CONASS: Conselho Nacional de Secretários de Saúde, Brazil’s National Council of Secretaries of Health
OECD: Organization for Economic Co-operation and Development
SDGs: Sustainable Development Goals
SUS: Sistema Unico de Saúde, Brazil’s Unified Health System
